# 637. Transplant Candidacy for Persons Living with HIV and End-Stage Renal Disease at MUSC

**DOI:** 10.1093/ofid/ofac492.689

**Published:** 2022-12-15

**Authors:** Kyle M Crawford, Ruth O Adekunle

**Affiliations:** Medical University of South Carolina, Charleston, South Carolina; Medical University of South Carolina, Charleston, South Carolina

## Abstract

**Background:**

Persons Living with HIV (PLWH) with chronic kidney disease progress to end-stage renal disease (ESRD) at a faster rate than HIV-negative individuals. Unfortunately, there is increasing data to suggest that PLWH are less likely to be waitlisted. We reviewed the transplant care continuum among PLWH at the Medical University of South Carolina (MUSC).

**Methods:**

This was a retrospective review of PLWH and ESRD who either received care at MUSC Health Care System or were referred for Kidney transplant at MUSC between May 1^st,^ 2012 and December 31^st,^ 2021. Cases were included if a PLWH and ESRD had not died before being referred for kidney transplant, had evidence of pursuit of kidney transplant, and sufficient data of their transplant process were available in the medical record system. Descriptive statistics were used to analyze demographic and clinical characteristics as well as the transplant care continuum of PLWH and ESRD at MUSC.

**Results:**

57 PLWH and ESRD were included in the analysis. Of these, 45 (79%) were referred for kidney transplant (Table 1). The median age at the time of referral was 52 (range 26 - 78), 33 (73%) were males and 42 (93%) were of the Black race. The most common causes of ESRD were Multifactorial (36%), HIV-Associated Nephropathy (24%) and unknown (16%). Almost all patients had hypertension (96%) and 29% of patients had diabetes. Additionally, prevalent comorbidities include psychiatric conditions (53%), history of substance use (43%) and history of tobacco use (36%). Of those referred for kidney transplantation, 32 (71%) started a transplant evaluation, 24 (53%) completed the transplant evaluation, 22 (49%) were waitlisted, and 13 (29%) were transplanted. Common reasons why patients did not complete transplant evaluation included not meeting HIV criteria for transplant (9%), having too many comorbidities (9%), and not completing testing (9%).

Clinical Characteristics of the 45 HIV Patients with ESRD Who Were Referred to Kidney Transplant at MUSC

**Figure:**
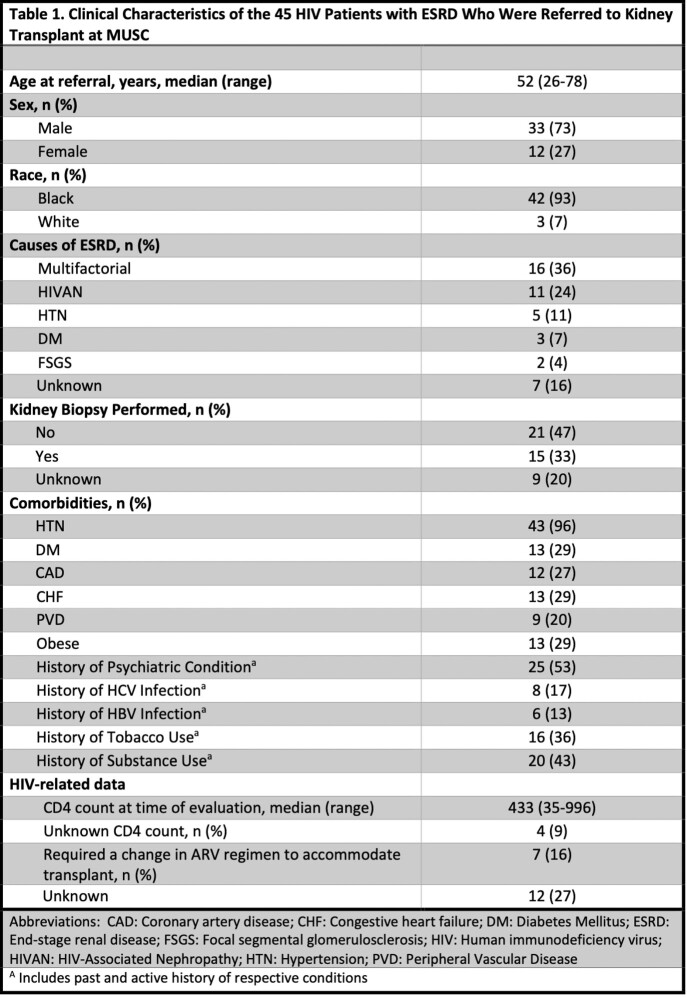

Clinical Data on Transplant Care Continuum, n = 45

**Figure:**
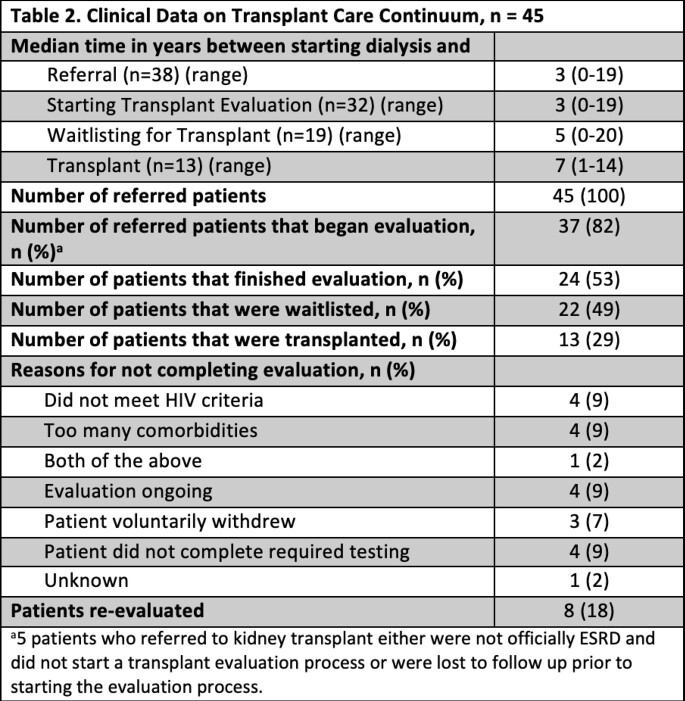

**Conclusion:**

The majority of PLWH with ESRD seen at MUSC were referred for kidney transplant and started the evaluation process. More is needed, though, to assist patients in completing the transplant evaluation process and making it to the transplant waitlist, with the ultimate goal of transplantation.

**Disclosures:**

**All Authors**: No reported disclosures.

